# ISG15 overexpression compensates the defect of Crimean-Congo hemorrhagic fever virus polymerase bearing a protease-inactive ovarian tumor domain

**DOI:** 10.1371/journal.pntd.0008610

**Published:** 2020-09-15

**Authors:** Stephanie Devignot, Thilo Kromer, Ali Mirazimi, Friedemann Weber

**Affiliations:** 1 Institute for Virology, FB10-Veterinary Medicine, Justus-Liebig University, Giessen, Germany; 2 Faculty of Health, Safety, Society, Furtwangen University, Furtwangen, Germany; 3 Public Health Agency of Sweden, Solna, Sweden; 4 National Veterinary Institute, Solna, Sweden; 5 Karolinska Institute, Stockholm, Sweden; NIAID Integrated Research Facility, UNITED STATES

## Abstract

Crimean-Congo Hemorrhagic Fever virus (CCHFV; family *Nairoviridae*) is an extremely pathogenic member of the *Bunyavirales* order. Previous studies have shown that the N-terminal domain of the CCHFV polymerase (L) contains an ovarian tumor-type protease (OTU) domain with the capability to remove both ubiquitin and ISG15 molecules from proteins. The approximately 200 amino acids-long OTU domain, if ectopically expressed, can interfere with both the induction of antiviral type I interferons (IFN) as well as the IFN-stimulated signaling. A OTU protease mutant (C40A), by contrast, was inactive in that respect. However, the effect of the OTU protease activity in the context of the full-length L protein (approximately 4000 amino acids) is only poorly characterized, and recombinant CCHFV with the C40A mutation could not be rescued. Here, we employed transcriptionally active virus-like particles (tc-VLPs) to investigate the interaction between the L-embedded OTU protease and the IFN system. Our data show a *cis* requirement of the OTU protease for optimal CCHFV polymerase activity in human HuH-7 cells. The L-embedded OTU did not influence IFN signaling, the sensitivity to IFN, or IFN induction. Moreover, the attenuation of OTU C40A-mutated L could not be relieved by inactivating the IFN response, but after overexpression of conjugation-competent ISG15 the polymerase activity recovered to wild-type levels. Consequently, ISG15 was used to produce OTU-deficient tc-VLPs, a potential vaccine candidate. Our data thus indicate that in the context of full-length L the OTU domain is important for the regulation of CCHFV polymerase by ISG15.

## Author summary

Tick-transmitted Crimean-Congo Hemorrhagic Fever virus (CCHFV) causes a serious and potentially fatal disease in humans. The CCHFV polymerase possesses an N-terminal ovarian tumor-type protease (OTU) domain that cleaves ubiquitin and ISG15 modifiers from target proteins. Previous studies demonstrated that the ectopically expressed OTU domain can inhibit antiviral type I interferon responses. Hence, cleavage-negative OTU mutants of virus or transcriptionally active virus-like particles (tc-VLPs) are expected to exhibit elevated immunogenicity and would be candidates for a live vaccine. For unknown reasons, however, recombinant virus with just the OTU minus mutation cannot be generated. Using tc-VLPs, we show that in human HuH-7 cells the activity of the OTU minus polymerase is reduced by more than 80%. Curiously, the attenuation could not be compensated by inactivating the interferon system or by adding the OTU domain *in trans*. However, a complete reversion of the OTU minus phenotype was achieved by transcomplementation with ISG15, whereas the other OTU substrate, ubiquitin, had no such positive influence. Our data thus indicate a role of *cis* OTU in CCHFV polymerase regulation that is independent of an anti-interferon activity but connected to ISG15. Transcomplementation with ISG15 may be a means to rescue the OTU minus CCHV vaccine candidate.

## Introduction

Crimean-Congo hemorrhagic fever virus (CCHFV) is a tick-transmitted member of the order *Bunyavirales* (family *Nairoviridae*, genus *Orthonairovirus*) that is present in endemic spots all over Africa, Asia, as well as South and East Europe [1, 2]. While CCHFV is able to infect a wide range of domestic and wild animals including bats [3], humans and primates are the only known host species that develop severe disease [1]. Symptoms of human CCHFV infection range from a mild febrile illness to severe hemorrhagic disease, with case fatality ratios between 5% and 30% [1]. Despite the severity and geographical range of the disease, there are no approved vaccines or treatments available.

CCHFV particles are spherical with a diameter of 80 to 100 nm [1]. On the outside, the lipid envelope displays spikes of the glycoproteins (GPs) Gn and Gc. Inside, the virions contain three different nucleocapsids (ribonucleoprotein particles; RNPs), each consisting of a negative-stranded RNA segment (vRNA) encapsidated by the nucleocapsid protein N and associated with the RNA-dependent RNA polymerase (RdRP), L. With an overall size of approximately 19.000 nt altogether, the three-segmented genome is among the largest within the group of negative-strand RNA viruses. Each genome segment contains one reading frame that is flanked by untranslated regions (UTRs) at the 3’ and 5’ ends, serving as promoters for mRNA transcription and genome replication [4]. The L segment encodes the L RdRP, the M segment a polyprotein encompassing Gn, Gc, some secreted proteins and a non-structural protein (NSm), and the S segment encodes the N protein and a non-structural protein (NSs) [1, 5, 6].

Viral docking and entry of RNPs into cells are mediated by the envelope GPs in a pH-dependent manner [7]. Once inside the cytoplasm, the RNP-associated L RdRP transcribes the encapsidated genome segments into mRNAs (primary transcription). The newly synthesized L and N proteins then generate an encapsidated full-length copy of the vRNA, the cRNA. The cRNA serves as template for the synthesis of vRNAs that on one hand are template for secondary mRNA transcription and on the other hand are packaged as RNPs into progeny virions [1, 5, 6].

The causes of the extreme symptoms by CCHFV in humans are not characterized. However, one factor that may contribute to pathogenesis is the ability to counteract innate immune responses, especially those mediated by the type I interferon (IFN-α/β) system [8]. Type I IFNs are cytokines that are produced as an alarm signal by infected cells. To achieve this, viral hallmark structures (e.g. double-stranded RNA, dsRNA) need to be sensed by cellular pathogen recognition receptors, which then trigger a specific signaling chain. This so-called “antiviral signaling” culminates in the activation of transcription factors such as IRF3 and NF-κB that bind to the promoters of IFN-β and other cytokines [9, 10]. After mRNA translation and secretion, the IFNs are docking onto their cognate receptor (IFNAR) to activate transcription of IFN-stimulated genes (ISGs) via the so-called JAK/STAT signaling pathway. Of the > 1000 known ISGs [11], many encode immunomodulatory or antiviral proteins. Among the important ISG products are the human MxA, which was shown to inhibit CCHFV infection [12], and ISG15, a small ubiquitin-like protein modifier that regulates the IFN response [13].

CCHFV is sensitive to the antiviral state if IFN is given to cells before infection [14], but IFN added on top of already infected cells is not inhibitory anymore [15]. Moreover, CCHFV significantly delays IRF3 activation and IFN induction [15]. In line with these observations, several strategies of the virus to counteract the IFN system were uncovered [6, 16]. Firstly, CCHFV processes the triphosphate (5’ppp) group on the 5’ end of its RNA genome to a 5’monophosphate [17]. The presence of 5’ppp-bearing RNAs is a widespread hallmark of virus infection, triggering IFN induction via the pathogen recognition receptor RIG-I [10, 18]. By removing the 5’ppp, CCHFV largely reduces IFN induction elicited by the genomic RNA [17, 19]. Moreover, the N-terminal domain of the CCHFV L RdRP contains a protease domain of the OTU (ovarian tumor) family that has the capability to remove both ubiquitin and ISG15 molecules from target proteins [20, 21]. Such a deubiquitinating (DUB) activity was also described for other nairoviruses as well as for arteriviruses and plant-pathogenic tymoviruses [22, 23].

Posttranslational protein modification by attachment of ubiquitin or ISG15 is an important regulatory mechanism of innate immunity [13, 24, 25]. In line with this, ectopic expression of viral OTU domains can interfere with the signaling triggered by IFN, RIG-I and NF-κB, and antagonize the antiviral effect of ISG15 [20, 26–28]. However, for CCHFV it was shown that the transiently expressed OTU domain locates to the cell nucleus, whereas longer fragments or the 3.945 aa full-length L are cytoplasmic [26, 29]. Moreover, compared to the less than 200 aa short OTU domain, 1.800 amino acids long N-terminal nairovirus L fragments already exhibited a reduced ability to suppress IFN induction and IFN-driven responses, and have entirely lost the ability to suppress the NF-κB-pathway [26, 27]. Thus, it appears likely that a cloned OTU cDNA plasmid does not faithfully reflect the behavior of the OTU domain present on the full-length L. Nonetheless, a recombinant CCHFV with an inactivated ubiquitin binding site of the OTU exhibited an elevated IFN induction compared to a wt recombinant and was attenuated in IFN-competent cells [30]. Thus, at least IFN induction under virus infection is modulated by the OTU of full-length L, an activity that is connected to ubiquitin binding.

While the results from the overexpressed OTU domain are difficult to interpret for the above-mentioned reasons, also for OTU in the viral context several questions have remained open. Firstly, it is unclear if the ability to decrease IFN induction is due to an active suppression (as it was shown for the isolated OTU domain), or if it is caused by a tight regulation of the RdRP to avoid the production of dsRNA or other pathogen recognition receptor-activating side products. In fact, the mentioned ubiquitin binding-deficient CCHFV mutant produces elevated RNA levels in some cell lines, whereas another virus mutant, deficient in both ubiquitin and ISG15 binding, produced less RNA than wt CCHFV (and was a poor IFN inducer [30]). Inactivating the OTU protease activity, by contrast, resulted in a lethal phenotype, i.e. such a mutant could not be rescued from cDNA, except if ubiquitin binding was additionally destroyed [30, 31]. Thus, the protease-negative L mutant of CCHFV, mutated exclusively in the catalytically active site of the OTU (C40A), could not be studied in the viral context.

In order to address the importance of the OTU protease activity for the function of the CCHFV L protein, we employed our system to generate transcriptionally competent virus-like particles (tc-VLPs) [32]. tc-VLPs are produced from transfected cDNA plasmids, contain a reporter minigenome instead of a viral genome, and are able to infect cells to transcribe and replicate their encoded reporter gene. In contrast to virus infection, in VLP systems the viral proteins are generated from the transfected plasmids, i.e. progeny particle production is uncoupled from the viral polymerase activity. Using this system, we circumvented the lethal phenotype of the protease-negative C40A OTU mutant and investigated the interplay between the L-embedded OTU protease with the IFN system, ubiquitin and ISG15. Our results indicate a positive regulatory role of ISG15 turnover on the CCHFV RdRP activity.

## Methods

### Cells and viruses

HuH-7, A549, Vero E6 (all kindly provided by Stephan Becker, Marburg, Germany), BSR T7/5 cells (kindly provided by K.-K. Conzelmann (Max-von-Pettenkofer Institute, Munich, Germany [33])) were grown in Dulbecco’s modified Eagle’s medium (DMEM) supplemented with 10% fetal calf serum (FCS), 2 mM glutamine, 100 U/ml penicillin, and 100 μg/ml streptomycin. For the BSR-T7/5 cells, medium was also supplemented with 5% tryptose phosphate broth (Sigma aldrich), 1% non-essential amino acids, and freshly added 2% geneticin. SW-13 cells (kindly provided by Pieter Leyssen, Rega Institute, Leuven, Belgium) were grown in Rega 3 minimum essential medium (MEM), supplemented with 10% FCS, 2 mM glutamine, 100 U/ml penicillin, 100 μg/ml streptomycin and 7.5μg/mL sodium bicarbonate.

Crimean-Congo hemorrhagic fever virus (CCHFV) strain IbAr10200 was propagated on SW-13 cells under BSL-4 conditions (Institute for Virology, Marburg, Germany). Vesicular stomatitis virus (VSV) was propagated on Vero E6. Medium and supplements are from Thermo Fisher Scientific, except mentioned otherwise.

### Plasmids

The constructs encoding wild-type, OTU inactive, and RdRp inactive CCHFV L polymerases (pCAGGS_V5_L_wt, pCAGGS_V5_L_C40A, pCAGGS_V5_L_ΔDD), CCHFV N nucleoprotein (pCAGGS_N), CCHFV glycoproteins (pCAGGS_GP), and the CCHFV S-specific Gaussia luciferase minigenome (V(0.0)_vS_Gluc(1G)), kindly provided by Eric Bergeron and Stuart Nichol (CDC Atlanta), as well as the CCHFV L-specific Renilla luciferase (Ren-Luc) minigenome (pT7riboSM2_vL_Ren), the T7 polymerase expression construct (pCAGGS_T7), the negative control (pcDNA3.1_3×Flag_ΔMx), and the empty vectors (pcDNA5/FRT/TO, pI.18 and pCAGGS) were described previously [32, 34–37]. The firefly luciferase (FF-Luc) reporter plasmids p125-FF-Luc (IFN-beta promoter) and pGL3-Mx1P (mouse Mx1 promoter) were kind gifts from Takashi Fujita (Laboratory of Molecular and Cellular Immunology, Kyoto, Japan) and Georg Kochs (Institute of Virology, Freiburg, Germany), respectively [38, 39]. The plasmids pGL3-luc and pRL-SV40, constitutively expressing FF-Luc or Ren-Luc respectively, were purchased from Promega.

The following plasmids were made by conventional cloning, using Phusion High-fidelity DNA polymerase (Thermo Fisher Scientific), primer sequences are available upon request.

The plasmids pI.18_OTU and pI.18_OTU_C40A, encoding the wild-type OTU or enzymatically-inactive mutant OTU_C40A domains (amino acids 1–169 of the CCHFV polymerase L), were subcloned from pCAGGS_V5_L_wt and pCAGGS_V5_L_C40A, respectively. To clone the plasmids pcDNA5/FRT/TO-USP18 (encoding a triple-FLAG version of USP18) and pcDNA5/FRT/TO-ISG15 (encoding an untagged version of pro-ISG15), RNA was first extracted from IFN-stimulated cells and then reversed transcribed into cDNA which was subsequently used as template for the PCRs. The plasmids pcDNA5/FRT/TO-ISG15act (encoding the active form of ISG15, ending by the RLRLGG motif) and pcDNA5/FRT/TO-ISG15act_ΔGG (encoding the active but non-conjugatable form of ISG15, ending by a RLRL motif) were created using pcDNA5/FRT/TO-ISG15 as template. The pCMV-His-Ubiquitin was a generous gift from Dr. Rita Moreno (Giessen, Germany).

### Real-time RT-PCR

VLPs were produced as described above, and proper production was validated by luciferase measurement in both donor and indicator cells. Total RNA was extracted from donor cell lysates using RNeasy kit (Qiagen), and from Benzonase-treated donor cell supernatants using QIAamp Viral RNA Mini Kit (Qiagen).

RNA standard required for absolute quantification by qPCR was made as follows. Plasmid pT7-RiboSM2_vL-Ren (encoding negative sense Ren-Luc) was linearized by cleavage in 3’ of the Ren-Luc cDNA sequence, using restriction enzyme *Age*I. The plasmid was purified by Phenol-Chloroform-Isoamylalcohol extraction (Sigma) and over-night precipitation in 100% EtOH / 3M NaOAc. In vitro transcription was made by the T7 polymerase (NEB) for 2h at 37°C. The RNA was analysed on gel (6% Urea-Page in TBE buffer), purified as above, and quantified.

Samples and RNA standard were reverse-transcribed using the PrimeScript RT Reagent Kit with gDNA Eraser (Takara), and a strand-specific Ren-Luc primer (Renilla-F, AACGCGGCCTCTTCTTATTT).

Sample cDNAs and standard cDNA (serial diluted and in triplicates) were subjected to quantitative PCR using TB Green Premix Ex Taq (Tli RNase H Plus) (Takara), and Ren-Luc-specific primers (Renilla-F, AACGCGGCCTCTTCTTATTT; and Renilla-R, ACCAGATTTGCCTGATTTGC), in a StepOne plus instrument (Applied Biosystems). Data were analysed as absolute quantification in RNA copies per mL of lysate buffer or per mL of supernatant.

### CCHF minireplicon system

The protocol for the CCHF minireplicon system was adapted from Bergeron *et al*. [34]. Subconfluent monolayers of BSR T7/5 seeded in 12-well plates were transfected with 600 ng of pCAGGS_V5_L (wt, or C40A, or ΔDD), 200 ng of pCAGGS_N, 200 ng of V(0.0)_vS_Gluc(1G), 200 ng of pCAGGS_T7, and 100 ng of pGL3-Luc control, using jetPRIME transfection reagent (Polyplus transfections). Four hours post-transfection (pt), the transfection medium was replaced with fresh medium. Cell supernatants were discarded 48h pt, and G-Luc and FF-Luc activities were measured in cell lysates using the dual-luciferase reporter assay system (Promega) and a Berthold TriStar2 LB942 luminometer with injector system. Results were first expressed as a ratio on the background level (condition using the RdRp-inactive mutant LΔDD) and then as a percentage compared to the wt L condition.

### Production of transcriptionally-competent virus-like particles (tc-VLPs)

The protocol to produce CCHF tc-VLPs was described previously [32]. 6-well plates with subconfluent monolayers of HuH-7 cells (“donor cells”) were transfected with 600 ng of pCAGGS_V5_L (wt, or C40A, or ΔDD), 200 ng of pCAGGS_N, 200 ng of pT7riboSM2_vL_Ren, 500 ng of pCAGGS_GP, 500 ng of pCAGGS_T7, and 25 ng of pGL3-Luc control, using GeneJammer transfection reagent (Agilent). At 72 h post-transfection, supernatants were harvested, and cells were lysed to measure Ren-Luc and FF-Luc activities as described above. The supernatants were cleared by centrifugation at 12,000 g for 5 min, and treated with 25 U/ml Benzonase (Novagen) at 37°C for 3 h. The presence of tc-VLPs was monitored using HuH-7 “indicator” cells. Indicator cells were first transfected with 600 ng pCAGGS_V5_L (wt or C40A) and 200 ng pCAGGS_N, incubated overnight, washed with phosphate-buffered saline (PBS), and then incubated with the harvested donor cell supernatants for 1 h at 37°C, after which fresh medium containing FCS was added. Reporter activities were measured 20 h later as described above. Results in donor cells were first expressed as a ratio on the background level (condition using the RdRp-inactive mutant LΔDD) and then as a percentage compared to the wt L condition. Results in indicator cells were calculated the same way, using the wt L condition in donor cells as reference.

### Immunoblotting of VLP donor cells content

For detection of CCHFV L polymerase (448kDa), lysate samples from tc-VLP donor cells were run through Criterion XT Tris-acetate precast gels (3–8% gradient) (Biorad) in a XT tricine buffer (Biorad), for 1 h 40 min at 150V. Proteins were transferred on an EtOH-activated polyvinylidene fluoride (PVDF) membrane (Millipore) by wet blotting over-night at 40 mA and 4°C, using Tris-glycine transfer buffer (5.8 g/L Tris (Acros), 2 g/L glycine (Roth), 10% absolute EtOH (Roth)). The CCHFV L was detected via its V5-tag (1:5000) (Novex, Life-technologies) and a horseradish peroxidase (HRP)-conjugated goat anti-mouse antibody (Thermo Fisher) (1:20,000). Quantification of the signals was performed using the Image Lab 4.0 software.

For detection of CCHFV Gn and N in donor cell supernatants, tc-VLPs (and CCHFV strain IbAr10200 as control) were produced, concentrated by ultracentrifugation through a 20% sucrose cushion, and their protein content analyzed by Western blotting (10% SDS-PAGE) and chemiluminescence quantification as described in [32].

All quantification values are shown as a percentage of the signal in wt L donor cells.

### Production of tc-VLPs in presence of IFNAR neutralizing antibodies or JAK1/2 inhibitor

tc-VLPs were produced as described above and treatment was done as follows. To block the interferon alpha receptor (IFNAR), the medium of the donor cells was replaced at 4 h post-transfection by fresh medium with or without 1μg/mL of neutralizing antibody against human interferon alpha/beta receptor chain 2 (CD118) (PBL). To block interferon signalling, donor cells were pre-treated for 2 h with 1μM JAK1/2 inhibitor Ruxolitinib (Selleckchem) or DMSO as control. Transfection of the donor cells was done in presence of the inhibitor or DMSO, and 4 h post-transfection the medium was replaced by fresh medium with ruxolitinib or DMSO.

### Interferon induction reporter gene assay

RNA from Vesicular stomatitis virus (VSV) particles was used to stimulate type I interferon induction. VSV was propagated on Vero E6 cells, and supernatant was collected 5 days post-infection. Particles were concentrated by PEG precipitation [32] and resuspended in TriFAST (VWR). RNA was extracted by addition of chloroform, and over-night precipitation of the upper aqueous phase with glycogen and isopropanol at -20°C. Pellets were washed twice with 70% EtOH and resuspended in TE buffer.

For the IFN induction reporter assay, subconfluent monolayers of A549 cells grown in 12-well plates were transfected with 150 ng p125-FF Luc (encoding FF-Luc under control of the IFN-β promoter) and 15 ng pRL-SV40 transfection control (encoding a constitutively expressed Ren-Luc), using GeneJammer (Agilent). The CCHFV OTU cDNA sequence was co-expressed either in the polymerase context (600 ng pCAGGS_V5_L_wt or pCAGGS_V5_L_C40A) or as isolated OTU domain (30 ng pI.18_OTU or pI.18_OTU_C40A). Amounts of full-length L and isolated OTU domain were adjusted to their relative sequence size. The total amount of transfected DNA was kept constant by adding the empty control vector (pI.18). At 24h post-transfection, the cells were transfected (JetPrime, PolyPlus-Transfections) with 250ng of RNA from VSV to stimulate the interferon pathway, or left unstimulated. Luciferase activities were measured 24 h later in cell lysates as described above.

### Interferon sensitivity assay

To assess the IFN sensitivity in the tc-VLP system, subconfluent monolayers of HuH-7 cells grown in 6-well plates were pre-transfected with 600 ng of pCAGGS_V5_L_wt and 200 ng of pCAGGS_N, using GeneJammer (Agilent). Twenty-four hours after transfection, medium was replaced either by untreated fresh medium or fresh medium containing 1,000 U/ml of recombinant human IFN-α B/D [40]. Cells were infected with tc-VLPs 20 h post-interferon, and Ren-Luc activity was measured 24 h post-infection.

To assess the IFN sensitivity in the minireplicon system, subconfluent monolayers of BSR T7/5 cells grown in 12-well plates were transfected with the CCHFV minireplicon plasmids. Twenty-four hours after transfection, medium was replaced either by untreated fresh medium or fresh medium containing 1,000 U/ml of recombinant human IFN-α B/D [40]. Ren-Luc activity was measured 44 h post-interferon.

### Interferon signaling reporter gene assay

Subconfluent monolayers of HuH-7 cells grown in 12-well plates were transfected with 150 ng pGL3-Mx1P (encoding FF-Luc under control of the Mx1 promoter) and 15 ng pRL-SV40 transfection control (encoding a constitutively expressed Ren-Luc), using GeneJammer (Agilent). The CCHFV OTU cDNA sequence was co-expressed either in the polymerase context (600 ng pCAGGS_V5_L_wt or pCAGGS_V5_L_C40A) or as isolated OTU domain (30 ng pI.18_OTU or pI.18_OTU_C40A). Amounts of full-length L and isolated OTU domain were adjusted to their relative sequence size in order to normalize for plasmid copy number. The total amount of transfected DNA was kept constant by adding a control vector (pcDNA3.13xFlag_ΔMx). Four hours after transfection, medium was replaced either by untreated fresh medium or fresh medium containing 1,000 U/ml of recombinant human IFN-α B/D [40]. Luciferase activities were measured 20 h later as described above.

### OTU transcomplementation assay and over expression of ubiquitin and ISG-15

HuH-7 donor cells and BSR-T7/5 cells, respectively for the tc-VLP and minireplicon systems, were transfected as described above, along with 200 ng pcDNA5/FRT/TO, pI.18_OTU, pI.18_OTU_C40A, pcDNA5/FRT/TO-USP18, pCMV-His-Ubiquitin, pcDNA5/FRT/TO-ISG15act, or pcDNA5/FRT/TO-ISG15act_ΔGG. In the tc-VLP system, indicator cells were pre-transfected with 600 ng pCAGGS_V5_L_wt and 200 ng pCAGGS_N, and then incubated with donor cell supernatants. Luciferase activities were detected as described previously, and values were expressed as a percentage of the wt L with control plasmid (empty vector).

### Passaging of tc-VLPs in the presence of ISG15

Subconfluent monolayers of HuH-7 donor cells seeded in T175 flasks were transfected with 4.7 μg of pCAGGS_V5_L (wt, C40A, or ΔDD), 1.5 μg of pCAGGS_N, 1.5 μg of pT7riboSM2_vL_Ren, 3.9 μg of pCAGGS_GP, 3.9 μg of pCAGGS_T7, and 1.5 μg of either the wild-type ISG15 (pcDNA5/FRT/TO-ISG15act) or the non-conjugatable ISG15act_ΔGG (pcDNA5/FRT/TO-ISG15act_ΔGG), using GeneJammer transfection reagent (Agilent). Cell supernatants were collected at 72 h post-transfection, cleared by centrifugation and treated with 25 U/mL Benzonase. Ren-Luc activities were measured in cell lysates and normalized and calculated as described below.

Passaging of the tc-VLPs was conducted as described previously [32]. Supernatants from donor cells were incubated onto new HuH-7 cells in T175 flasks, which were pretransfected 20 h earlier with 4.7 μg of pCAGGS_V5_L (wt, C40A, or ΔDD), 1.5 μg of pCAGGS_N, 3.9 μg of pCAGGS_GP, and 1.5 μg of either the wild-type ISG15 (pcDNA5/FRT/TO-ISG15act) or the unconjugatable ISG15_ΔGG (pcDNA5/FRT/TO-ISG15act_ΔGG). This was repeated for 2 passages.

At each passage, the presence of tc-VLPs was also assessed by incubation onto new HuH-7 cells in 6-well plates, which were pretransfected 20 h earlier with 600 ng of pCAGGS_V5_L_wt and 200 ng of pCAGGS_N. Ren-Luc activities were measured in cell lysates at 20 h post-infection and normalized to the values obtained with corresponding plasmid set containing the inactive L mutant ΔDD. In each experiment, data are expressed as a percentage of L wt / ISG15_ΔGG in donor cells (passage 0).

At each passage, tc-VLP were also titrated by immunofluorescence staining of Ren-Luc, as described previously [32].

### Statistics

Paired Student’s t tests were used to compare the different conditions with assumed variance equality (Levene > 0.05). Multiple comparisons were corrected using the Bonferroni method by multiplying the resulting p-values with the number of comparisons made within each data set. Corrected p-values were considered significant (*) if below 0.05.

## Results

### The L-associated OTU protease is important for polymerase activity in HuH-7 cells

**Fig 1:**
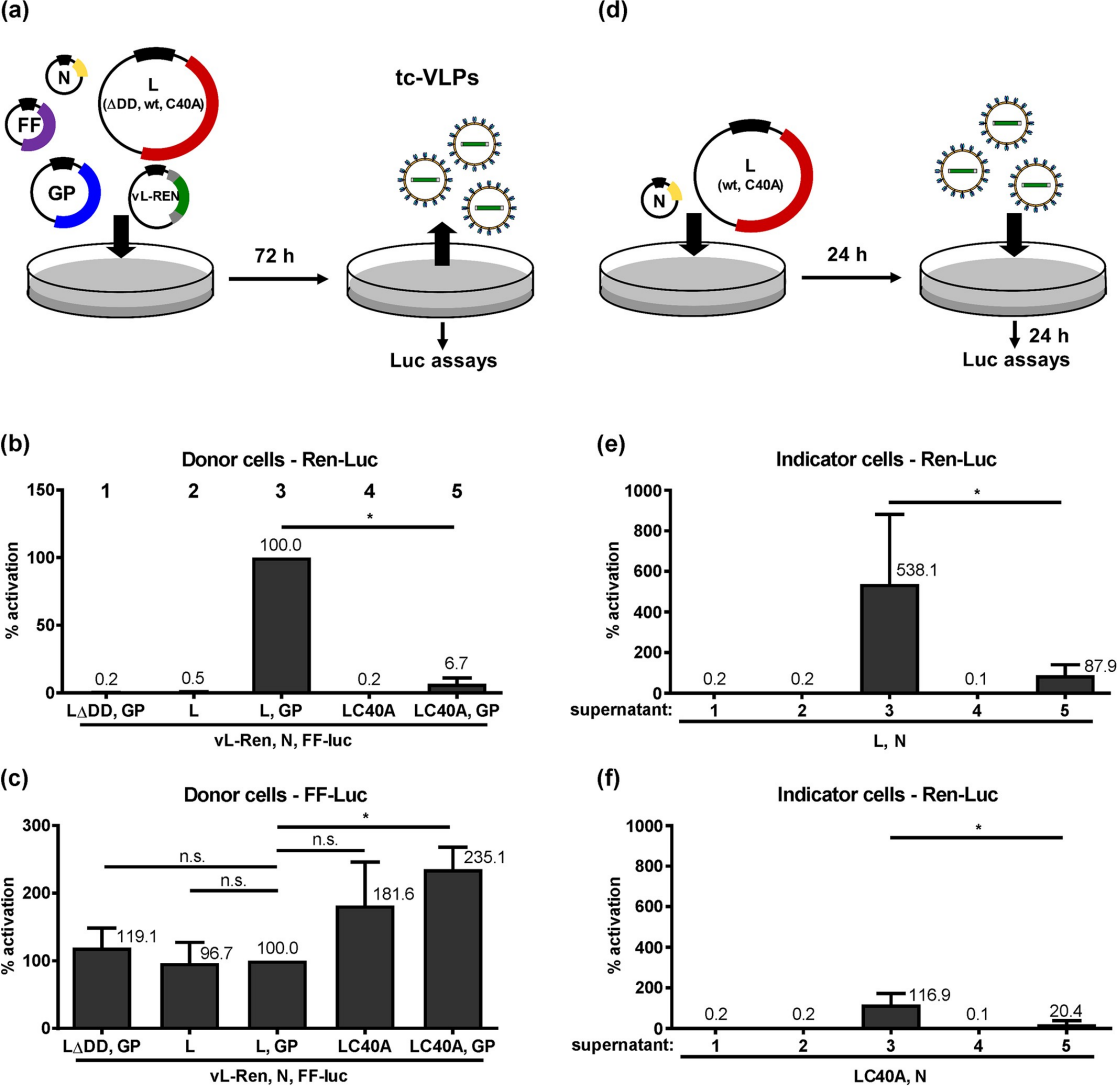
Catalytically inactive L OTU mutant C40A in transcriptionally active VLPs. (a) Cartoon depicting the experimental procedure of transfecting the tc-VLP donor cells. (b) Ren-Luc activity reflecting transcription and replication by the L RdRP. (c) FF-Luc activity reflecting general gene expression efficiency. (d) Cartoon depicting the experimental procedure of transfecting and infecting the tc-VLP indicator cells. (e and f) tc-VLP transcription and replication supported by expression of wt L RdRP (e) or the C40A L mutant (f) in indicator cells. All luciferase values are relative to the wt L VLP activity in donor cells, which was set to 100%. Mean values and standard deviations of 5 independent experiments are shown. Two-tailed, paired Student’s *t* tests without (b, e, f) or with Bonferroni correction for multiple comparisons (c) were used to compare the different conditions with the L,GP one. *, *P* < 0.05; n.s., not significant.

Using a minireplicon system consisting of transiently expressed CCHFV L, N and a reporter minigenome, it was found that the OTU protease-negative L mutant C40A exhibited the same RdRP activity as wt L [34] (see also below). On the other hand, it was not possible to generate a recombinant CCHFV bearing the C40A mutation in L, and data from tc-VLP-infected cells led Scholte *et al*. to conclude that C40A L impairs particle production and/or infectivity [30]. The minireplicon system studies [34] were undertaken in hamster BSR-T7/5 cells, whereas virus rescues and tc-VLP experiments were done with human HuH-7 cells [30]. Using our tc-VLP system [32], we investigated whether the C40A L mutation has an influence on particle production or infectivity. HuH-7 cells were transfected with plasmid constructs encoding CCHFV L (inactive mutant ΔDD, wt, or C40A), the nucleocapsid protein N, the viral GP, and a minigenome with a Renilla luciferase (Ren-Luc) reporter gene in negative-sense and under control of the L segment UTR promoter (vL-Ren) (Fig 1A). As internal expression and transfection control, we also transfected a plasmid encoding a firefly luciferase (FF-Luc) under control of a pol II-driven promoter. In the VLP-producing “donor cells”, expression of the wt L RdRP in the context of the full set of tc-VLP plasmids led to an approximately 15-fold higher activity than expression of the C40A mutant of L (Fig 1(B), columns 3 and 5). As observed before [32], omission of the GP-encoding plasmid rendered the system largely inactive, indicating that the minigenome system alone (consisting of L and N amplifying the vL-Ren minigenome) is inefficient in HuH-7 cells (Fig 1(B), columns 2 and 4). Of note, we consistently observed higher FF-Luc control activities upon L C40A RdRP expression compared to wt L (Fig 1(C)), indicating a general suppressive effect of the L-embedded OTU protease. When the VLP-containing supernatants of the donor cells were transferred onto HuH-7 “indicator” cells expressing N together with wt or C40A mutant L RdRP to amplify the minigenome carried over by the tc-VLPs (Fig 1(D)), a similar trend in minigenome reporter activity was apparent. tc-VLPs made with the wt L exhibited a much stronger reporter activity than tc-VLPs made with the OTU-deficient L C40A (Fig 1E and 1F). Moreover, also in indicator cells in which L C40A was used to support VLP activity, overall VLP activity was much weaker, independent of the RdRP type (wt or C40A L) within the infecting VLPs. Importantly, the activity difference between wt and C40A OTU L in donor cells is preserved (and not further increasing) in indicator cells. This suggests that the infectivity of mutant VLPs, once formed and released, is similar to the one of wt L VLPs.

**Fig 2:**
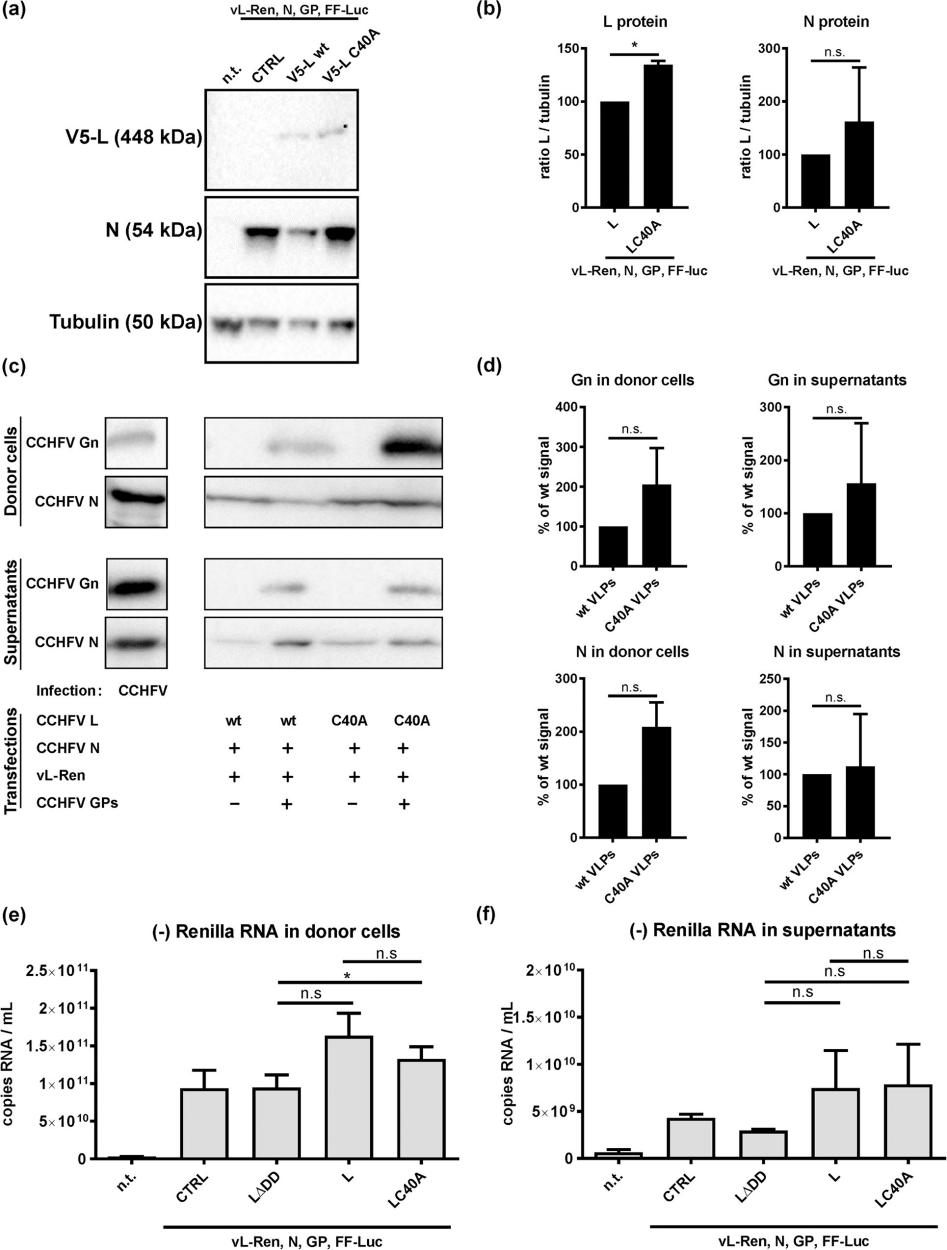
Levels of structural proteins and minigenome RNA. Cells were transfected as indicated for Fig 1. (a) and (b) Immunoblot analysis of lysates from tc-VLP donor cells. (a) Representative blots. (b) Quantification of immunoblot signals obtained by calculating the ratio of the respective L signal to tubulin and setting wt L to 100% (n = 3). (c) Immunoblot analysis of lysates from tc-VLP donor cells (top panels) and supernatants derived from them (bottom panels, representative blots shown). (d) Quantification of immunoblots as shown in (c) (n = 3). (e) and (f) Minigenome RNA synthesis. Total RNA from donor cells (e) and VLP-containing cell supernatants (f) were tested by negative-strand RNA-specific RT-qPCR for the Ren-Luc sequence. For each experiment, mean values and the standard deviations of 3 independent repeats are shown. Two-tailed, paired Student’s t test without (b, d) or with Bonferroni correction for multiple comparisons (e, f) were used. *, P < 0.05; n.s., not significant; n.t. non-transfected; CTRL, empty vector.

To measure whether the low L C40A VLP activity could be due to negative effects on protein levels, we performed immunoblot analyses. However, lysates from C40A tc-VLP donor cells showed increased, rather than decreased levels of L protein as compared to wt tc-VLP donor cells (Fig 2(A) and 2(B), left panel). In VLPs themselves, L levels were too low to be detected in the immunoblot (S1 Fig). However, levels of Gn (as representative of the GPs) both in tc-VLP donor cells and in the VLPs again did not decrease due to co-expressed C40A L (Fig 2(C) and 2(D), upper panel). For N, there was also no significant difference neither in donor cell lysates nor in VLPs (Fig 2(B), right panel and 2(D), lower panel).

Next we investigated whether the OTU activity may influence the production or packaging of L-generated minigenome RNA. Strand-specific RT-qPCR analyses of donor cells expressing no polymerase or the inactive LΔDD mutant detected the negative-sense RNA generated by the cotransfected Ren-Luc minigenome plasmid, as expected (Fig 2(E)). Co-expression of the active L variants wt L or LC40A slightly increased RNA levels above this background. However, there was no significant difference between the two active L variants. Similarly, amounts of negative-strand Ren-Luc RNA did not differ in VLP-containing supernatants harvested from either wt L or LC40A donor cells. (Fig 2(F)). These results do not support the hypothesis that the OTU domain is important for minigenome RNA replication or packaging. Thus, the C40A mutation of the OTU neither reduces L protein levels, nor VLP production or viral genome replication, but rather affects L-driven gene expression in HuH-7 cells.

### IFN sensitivity of CCHF-VLPs is independent of the OTU domain

**Fig 3:**
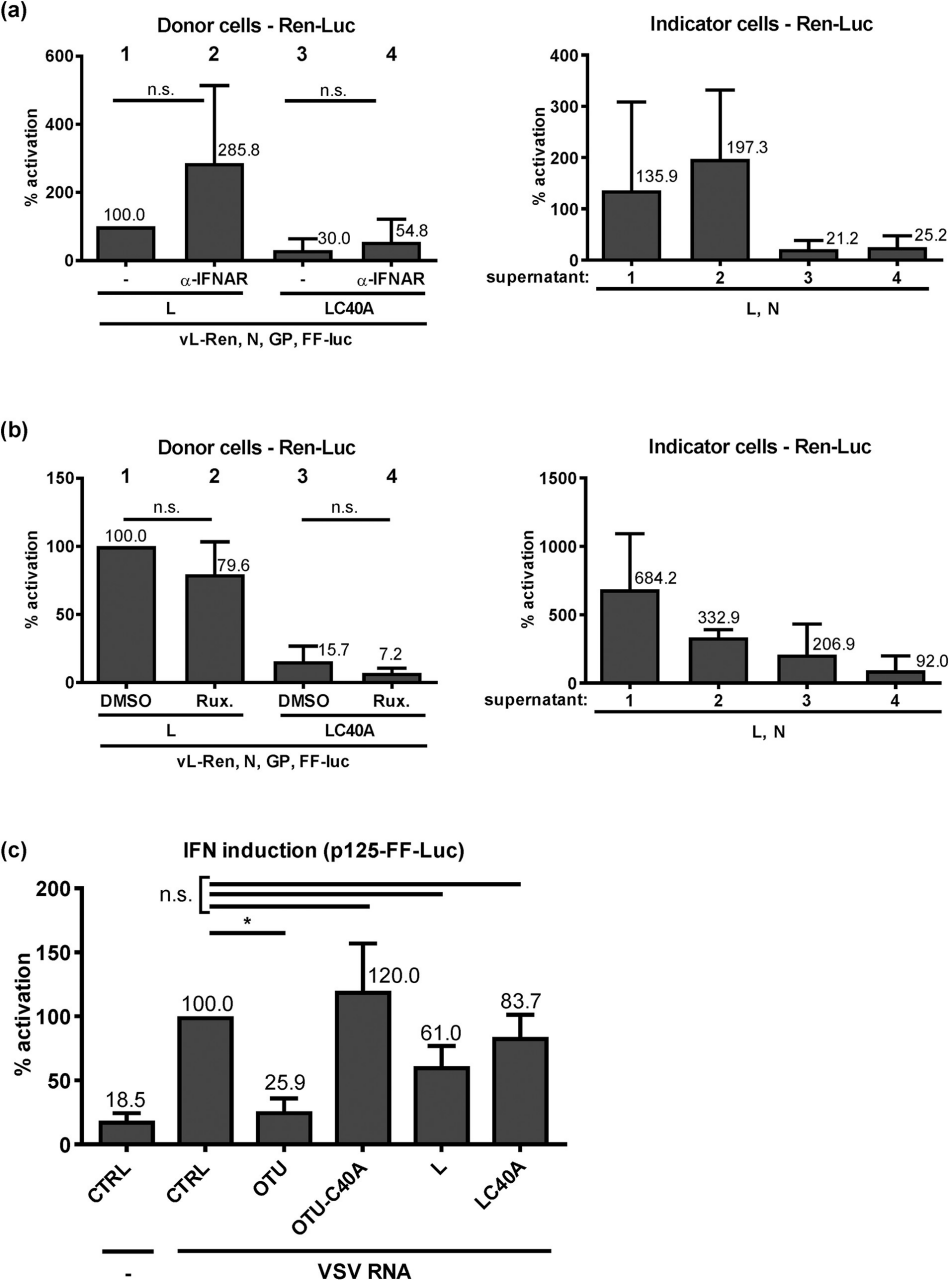
tc-VLP activity and IFN induction. (a) tc-VLP activity in donor cells (left) and indicator cells (right) in the presence of an antibody blocking the type I IFN receptor (IFNAR). (b) tc-VLP activity in donor cells (left) and indicator cells (right) under incubation with 1μM of the type I IFN signalling blocker Ruxolitinib (Rux). (c) Influence of overexpressed OTU or full-length L on IFN induction. A549 cells were pretransfected with expression constructs and the reporter plasmid p125-FF-Luc [39] containing the FF-Luc gene under control of the IFN-β promoter. Empty expression vector pI.18 was used as negative control (CTRL). The IFN-β promoter was induced by transfecting VSV particle RNA. Mean values and the standard deviations of 4 (a, b) or 3 (c) independent experiments are shown. Two-tailed, paired Student’s *t* tests without (a, b) or with Bonferroni correction (c) were used to test the indicated comparisons. *, *P* < 0.05; n.s., not significant.

In a minireplicon system, the C40A L mutant showed the same activity as wt L [34]. For those studies, however, hamster BSR-T7/5 cells were used that are known to have defects in IFN induction [35]. The systems in which C40A L exhibited an attenuated phenotype (virus rescues and tc-VLP experiments), by contrast, were based on human HuH-7 cells [30] (see above), which have a moderately active IFN system [41, 42]. Thus, it is possible that the OTU cleavage activity is required for RdRP activity in IFN-competent cell systems. For tc-VLPs of Rift Valley fever bunyavirus (RVFV), infection of naïve cells is sufficient to induce IFN gene expression [43]. In similar experiments with CCHFV tc-VLPs, however, we could not measure any specific IFN induction, irrespective of whether the tc-VLPs contained wt or C40A L RdRP (S2 Fig). Most probably this is due to several factors such as the absence of a RIG-I-triggering 5’ppp group in the genome of CCHFV [17] or the comparatively low activity of the OTU-deficient tc-VLPs. As an alternative, we tested whether inhibition of the IFN response could rescue the attenuated activity of the C40A L VLPs. However, neutralizing the IFN receptor with an antibody increased neither wt nor C40A tc-VLP activity in either donor cells (Fig 3(A), left panel) or indicator cells (Fig 3(A), right panel). Similarly, pretreating cells with the IFN response inhibitor Ruxolitinib, an established booster for IFN-inducing virus mutants [44, 45], had no positive effect on tc-VLP production or infection, again irrespective of the L genotype (Fig 3(B)). We also expressed the full-length L variants independently of the other viral genes, triggered IFN induction by an ectopically added elicitor RNA, and measured IFN induction by a reporter assay (Fig 3(C)). In this setting, the isolated OTU domain, but not any of the full-length L constructs, could suppress IFN induction. Taken together, two different measures to neutralize IFN failed to revert the attenuation of the OTU-deficient CCHFV RdRP in HuH-7 cells, and IFN induction antagonism by full-length wt L could not be observed. Thus, we confirm data obtained for the isolated OTU domain [20, 26–28], but cannot provide evidence that IFN induction would be the cause for the attenuation of the CCHFV RdRP with a catalytically inactive OTU domain.

**Fig 4:**
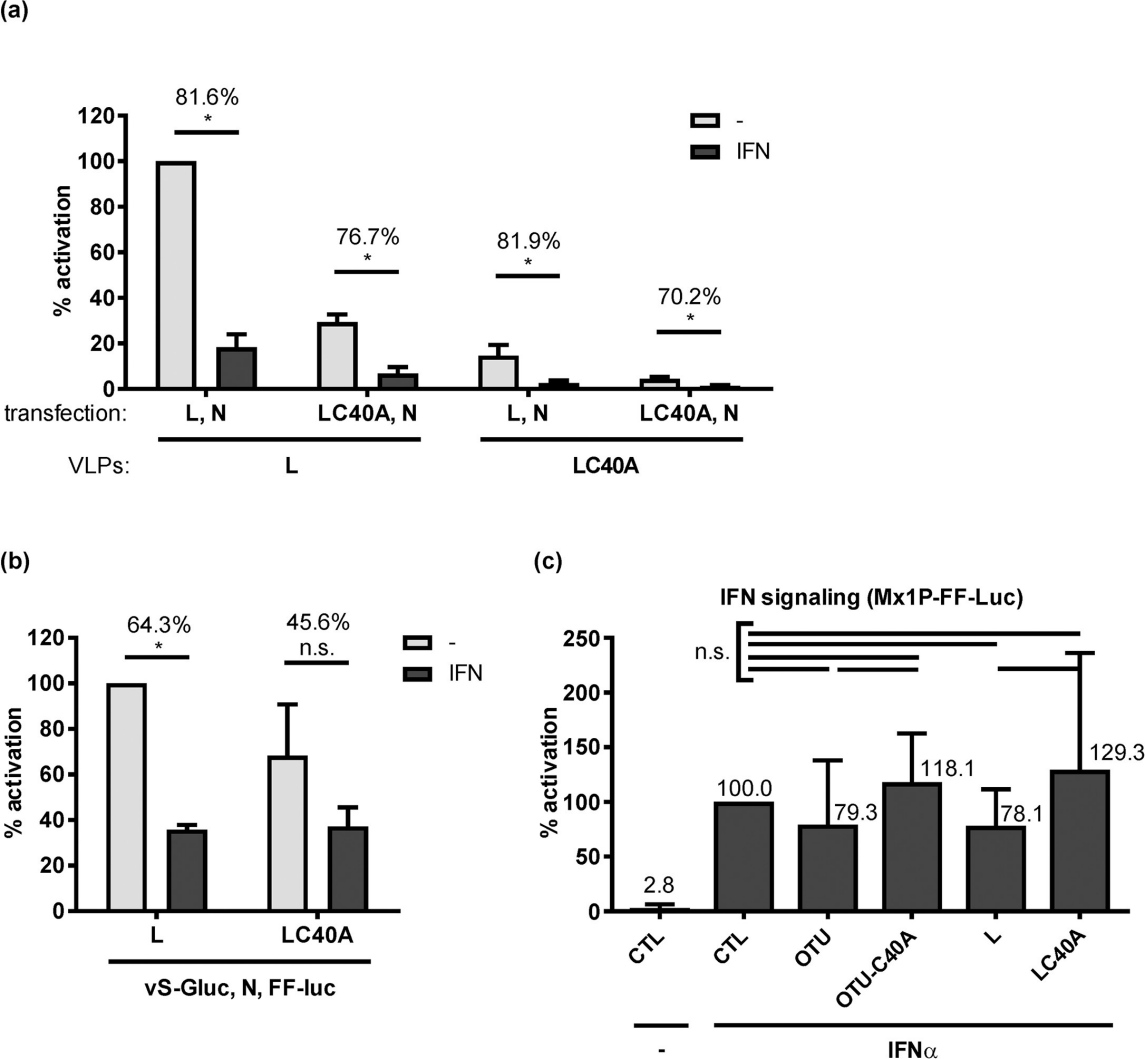
IFN effect on wt and C40A L. (a) tc-VLP activity in indicator cells. Cells were transfected with L and N support plasmids and incubated 24 h later with either medium (-) or medium containing 1,000 U/ml of IFN-α B/D (IFN). Combinations of wt and C40A mutant L were used to produce and support VLP activity in donor and indicator cells, as indicated. Infection with the L-tc-VLPs or the LC40A-tc-VLPs was performed 20 h post-interferon. (b) RdRP activity of wt and C40A L in the minireplicon system. BSR-T7/5 cells were mock treated or treated with 1000 IU/ml IFN at 24 h after transfection. Luciferase activities were measured 24 h post-infection (a) or 44 h post-interferon (b). Reporter values were normalized to the inactive L polymerase (LΔDD) condition that was transfected in parallel, and expressed as percentage of the wt L under untreated (-) condition. The percentage of reduction of luciferase activity between untreated and IFN-treated conditions is written on top of the histogram bars. (c) Influence of overexpressed OTU or full-length L on IFN signaling. HuH-7 cells were pretransfected with expression constructs and the reporter plasmid pGL3-Mx1P containing the FF-Luc gene under control of the IFN-responsive mouse Mx1 promoter [38]. IFN signalling was triggered by treating cells with 1,000 U/ml IFN at 4 h post-transfection and luciferase activities measured at 20 h post-transfection. Mean values and standard deviations of 3 (a, b) or 5 (c) independent experiments are shown. Statistical testing was performed as indicated for the previous figures. *, *P* < 0.05; n.s., not significant.

There remains the possibility that the polymerase-borne OTU influences the antiviral state imposed by IFN. In a first attempt to address this, we measured tc-VLP activity in cells which had been pretreated with IFN. As shown in Fig 4(A), 1000 IU/ml of IFN-α suppressed tc-VLP reporter activity by 70% or more. Importantly, this suppression level was again irrespective of whether VLPs or indicator cells contained wt L or the cleavage-inactive C40A mutant. Thus, both the IFN signaling (that could have been influenced by the L pretransfected into the indicator cells) and the IFN action (that acts on the infecting VLPs) are not influenced by the OTU activity. Moreover, we could not detect an elevated IFN sensitivity of the OTU mutant L in the CCHFV minireplicon system (Fig 4(B)), in which both wt and OTU mutant L were similarly active as expected [34]. Finally, we were also unable to detect any significant effect of the overexpressed CCHFV OTU domain or full-length wt L on IFN-stimulated ISG promoter activity, using an Mx1 reporter construct (Fig 4(C)). Thus, although the OTU C40A tc-VLPs clearly have an attenuated phenotype in HuH-7 cells, we could not find any evidence for an involvement of the antiviral IFN.

### *Cis* and *trans* activities of the OTU domain are different

**Fig 5:**
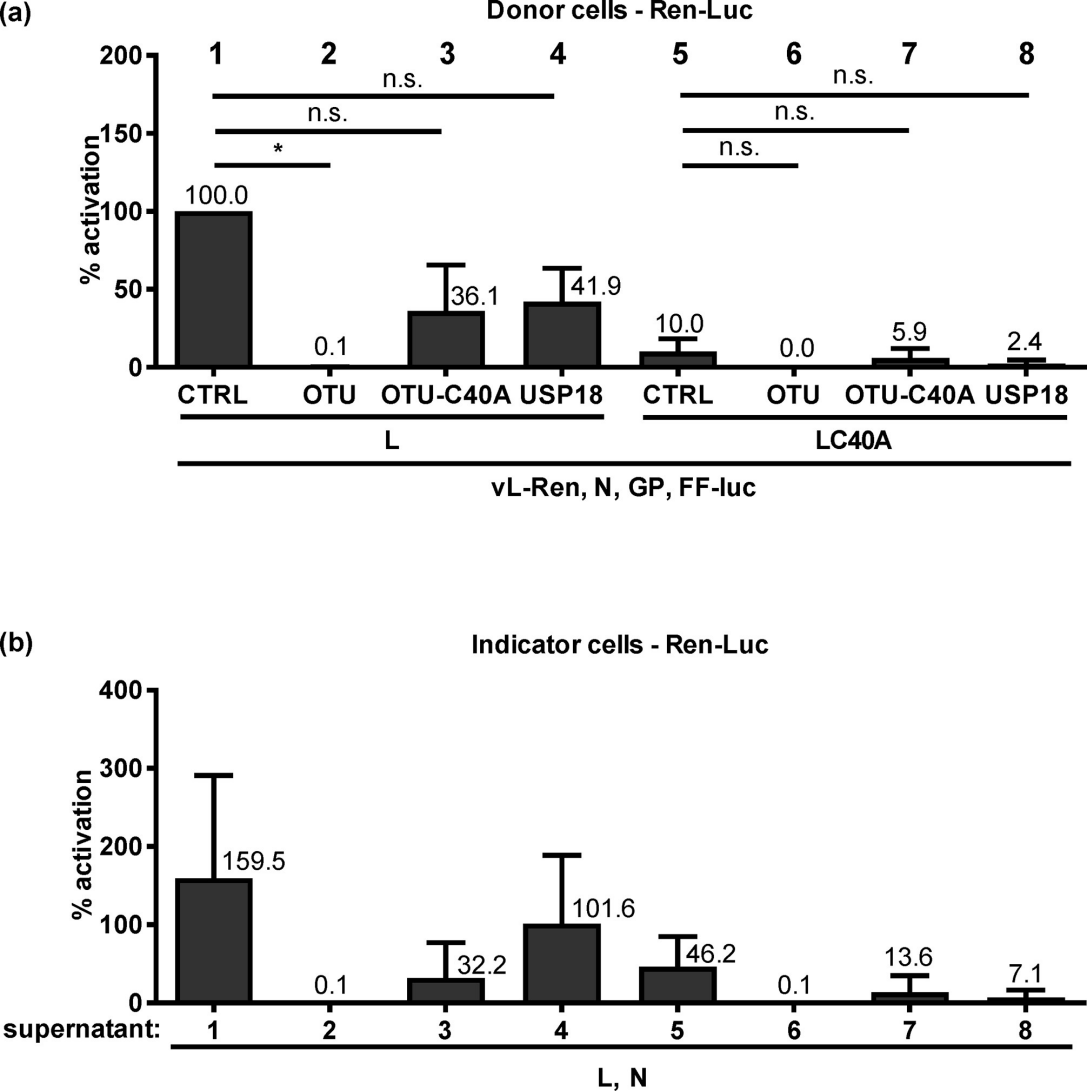
tc-VLP activity with OTU *in trans*. (a) Donor cells activity. Plasmids of the tc-VLP system encoding wt or C40A L were transfected together with expression plasmids for wt or C40A OTU domain, or for USP18. (b) Indicator cell activity mediated by the supernatants of (a). Mean values and the standard deviations of 3 independent experiments are shown. Two-tailed, paired Student’s *t* tests with Bonferroni correction were used to compare each CTRL with the different other constructs. *, *P* < 0.05; n.s., not significant.

We attempted to transcomplement the phenotype of the C40A L mutant by expressing the wt OTU domain (aa 1–169) together with the tc-VLP system. However, the wt OTU domain *in trans* could not rescue the weak reporter activity by the OTU-deficient L RdRP in the tc-VLP-producing donor cells (Fig 5(A)) or indicator cells (Fig 5(B)). The C40A OTU as well as the cellular de-ISGylation enzyme USP18 [46] were also not supportive. In fact, a negative influence of the isolated OTU domain, statistically significant for the wt L, was observed. Ectopically expressed OTU also suppressed L RdRP activity in minireplicon systems of CCHFV and RVFV, as well as just FF-Luc reporter activity (S3 Fig). This indicates a general, cell type- and virus-independent negative effect. We conclude that the OTU domain *in cis* (i.e. as part of the full-length L protein) increases RdRP activity in HuH-7 cells, whereas the isolated OTU domain *in trans* is an unspecific suppressor of gene expression.

### Conjugation-competent ISG15 compensates the attenuation of the cleavage-inactive C40A polymerase

**Fig 6:**
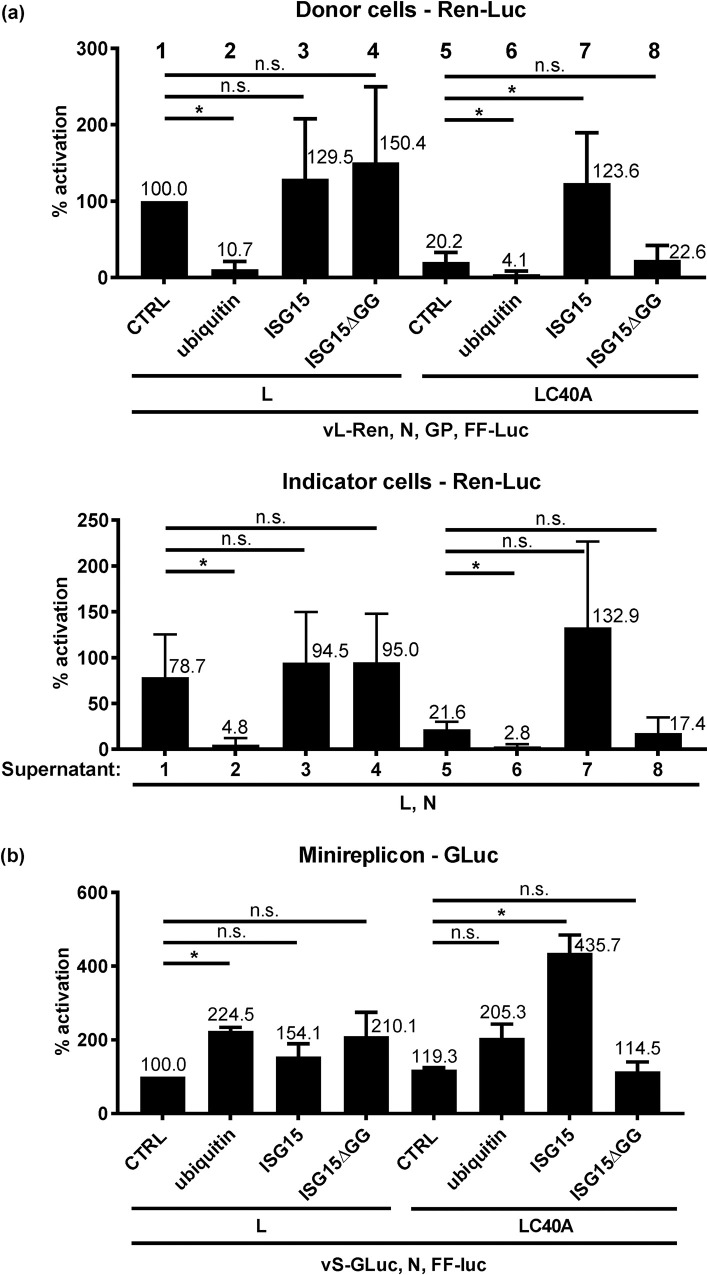
CCHFV RdRP activity with ubiquitin or ISG15 *in trans*. (a) tc-VLP activity. Plasmids of the tc-VLP system encoding wt or C40A L were transfected together with expression plasmids for ubiquitin, wt ISG15, the non-conjugatable ISG15 mutant ΔGG, or empty vector (CTRL). Top panel: activities in donor cells, bottom panel: activities in indicator cells. (b) Minireplicon activity. Plasmids with minireplicon system expressing wt or C40A L were cotransfected with a control plasmid (CTRL) or either ubiquitin (Ub) or ISG15 or ISG15_ΔGG. Mean values and standard deviations of 6 (a) and 3 (b) independent experiments are shown. Two-tailed, paired Student’s *t* tests with Bonferroni correction were used to compare the CTRL with the different other constructs. *, *P* < 0.05; n.s., not significant.

Since neither a suppression of the IFN system nor adding the OTU *in trans* could substantially rescue the attenuation of the C40A L RdRP, we investigated the effect of the OTU substrates, i.e. ubiquitin and ISG15. Overexpression of ubiquitin significantly suppressed the activity of both wt L and L C40A tc-VLPs (Fig 6(A), upper panel). However, overexpression of ISG15 increased L C40A tc-VLP activity back to wt L-like levels in donor cells, but had no effect on wt L. The ISG15-mediated boost in L C40A activity was dependent on the ability of ISG15 to be conjugated, as the deficient mutant ISG15ΔGG had no such effect. Transfer of the supernatants onto indicator cells suggested that also more LC40A VLPs were being generated in the presence of ISG15 (Fig 6(A), right panel), although significance levels were not reached in multiple testing (p = 0.084 in two-tailed, paired Student’s *t* tests with Bonferroni correction). The strong and specific positive effect of ISG15 on the L C40A polymerase could be confirmed in the CCHFV minigenome system (Fig 6(B)). Interestingly, in this system ubiquitin had a slightly increasing (rather than suppressing) influence on the wt L activity. Taken together, for L C40A the boosting effect of conjugatable ISG15 is on the L activity rather than on particle formation, and compensates (tc-VLPs) or even overcompensates (minireplicon system) for the loss of OTU activity.

### ISG15 overexpression enables higher yields of OTU-deficient tc-VLPs

**Fig 7:**
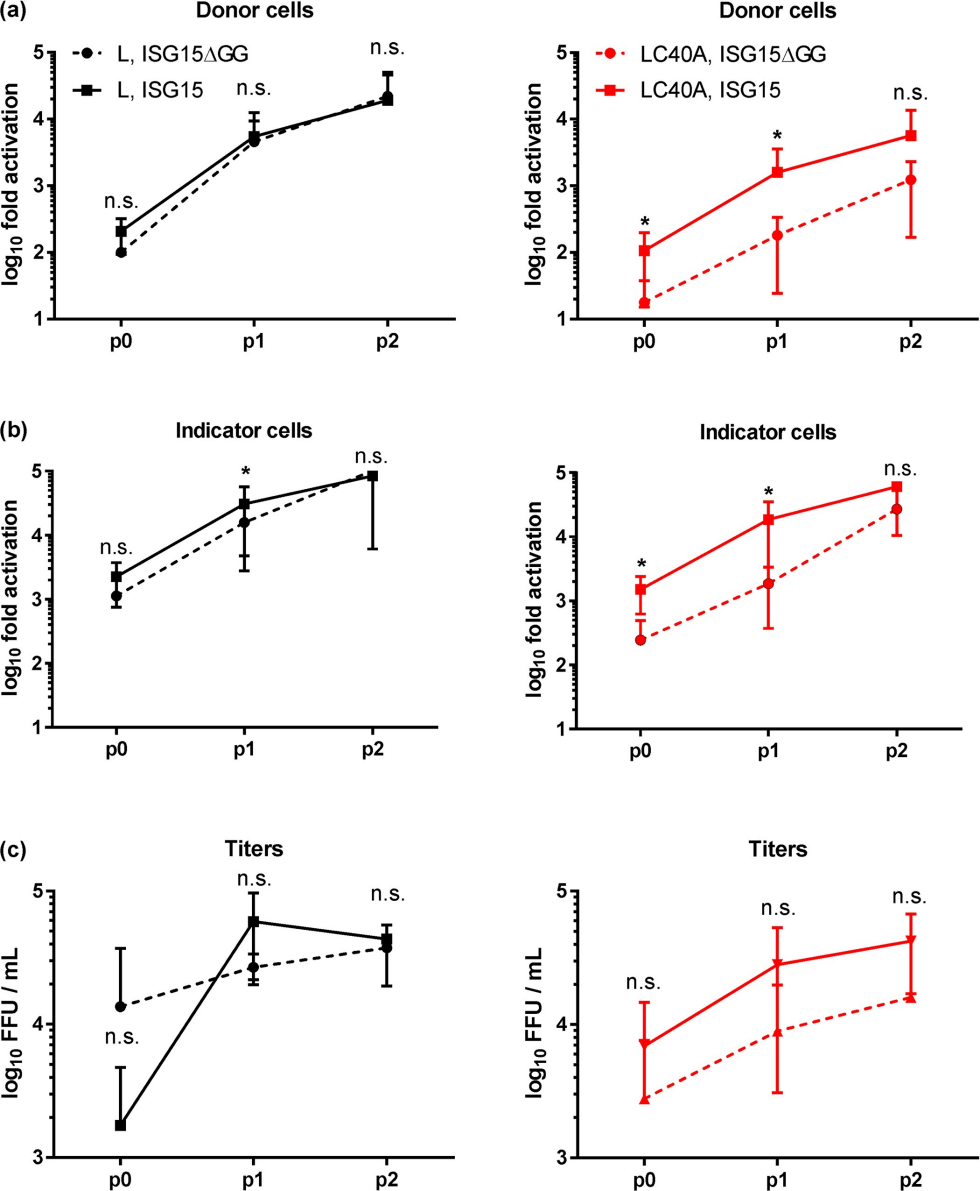
ISG15 as a possible booster of OTU mutant tc-VLP production. HuH-7 cells were transfected with the wt L or L C40A tc-VLP systems together with expression plasmids for wt ISG15 or the non-conjugatable ΔGG mutant (ISG15ΔGG). Ren-Luc activities of donor cells (a), indicator cells supported with wt L and N (b), as well as of VLP titers (c), are shown. p0, p1 and p2 designate the passages of VLPs on HuH-7 cells transfected with the corresponding L and ISG15 variants plus N. Ren-Luc activities were normalized first to the corresponding LΔDD/ISG etc. VLP mix which had been transfected in parallel, and then to values of wt L with the ISG15ΔGG negative control in p0 of donor cells. Mean values and standard deviations of 3 independent experiments are plotted. One-tailed, paired Student’s t test of log-transformed data was used to compare settings with ISG15ΔGG and wt ISG15 for each passage and each L variant. *, *P* < 0.05; n.s., not significant.

The catalytic domain of viral DUBs like the OTU is a preferred target for antiviral therapy [47, 48]. Moreover, viruses or tc-VLPs with catalytically inactive OTU domains may be prime candidates for live vaccines. Attempts to rescue a C40A mutated CCHFV have failed [30, 31], and the corresponding VLPs only reach low yields (see above). We took advantage of the complementation effect described above, and produced C40A mutant tc-VLPs in the presence of ISG15. Our earlier work showed that repeated passaging of tc-VLPs on cells expressing L, N and GP (but no minigenome) led to a stepwise increase in VLP production [32]. Now, when we added ISG15 to the plasmid mix in the passaging cells, the positive effect of conjugatable ISG15 over the ΔGG mutant ISG15 was maintained and tc-VLP activity with C40A L increased in passage 0 and after passage 1 (Fig 7(A) and 7(B)). With final passage 2, however, the difference between wt ISG15 and ΔGG ISG15 treatment was not significant anymore, suggesting a plateau has been reached. We had also determined VLP titers by manually counting Ren-Luc-positive indicator cells in the immunofluorescence. Also here the same trend were observed, but statistical significance was not reached, probably because this method produces standard deviations that are too high (Fig 7C). In contrast to the C40A L mutant, wt L tc-VLPs were not influenced by wt ISG15, with the notable exception of indicator cells after passage 1, in which ISG15 made a small but significant difference. Ectopic ISG15 expression may therefore be considered as an accelerator of C40A tc-VLP production, and probably also to help rescue the so far lethal phenotype of the virus mutant.

## Discussion

The OTU domain on the L protein of CCHFV is an antagonist of innate immunity, a function assumed to rely on its DUB and deISGylase activities against host cell factors [23]. However, while isolated OTUs of CCHFV and other nairoviruses do exhibit a strong and general capability to inhibit exogenously triggered IFN induction, IFN signaling, and TNF-α signaling [20, 22, 26–28, 49], longer N-terminal L fragments containing the wt OTU domain gradually lost their potency [26, 27]. The full-length CCHFV L RdRP was however not tested in that respect, and we show IFN induction is not inhibited to a statistically significant degree. Thus, our data suggest caution in interpreting data from experiments with isolated OTU. Moreover, both the OTU and the full-length CCHFV L appear to have a general negative effect on gene expression that needs to be taken into account.

Among the nairoviral OTUs there is great variability with respect to the DUB and deISGylase activities, with some species possessing only one of these functions, and some that are even entirely devoid of both [50]. CCHFV, being arguably the most pathogenic of the nairoviruses, has rather moderate DUB and deISGylase activities. For CCHFV and Dugbe nairovirus it was also shown that both DUB and deISGylation activities decrease with increasing L fragment length [20, 26]. Thus, although the OTU domain alone has a clear anti-IFN function and the DUB/deISGylase are well established biochemical properties, the connection between these activities may not be straightforward.

Here, we show that the CCHFV L polymerase mutant C40A, lacking the OTU protease activity, is attenuated in human cells, perhaps explaining the lethal phenotype of recombinant C40A virus [30, 31]. Although the L attenuation is absent in the IFN-deficient BHK cells, it could not be relieved by blocking the IFN system in human cells. Along the same lines, ectopically expressed wt OTU, able to suppress IFN induction by an ectopic elicitor RNA, could not transcomplement the attenuated phenotype. We therefore conclude that the attenuation of OTU-inactive full-length CCHFV L is not related to a loss of its anti-IFN activity, but might be involved in cell type or host-specific regulation of the polymerase. Our results thus indicate that the OTU protease domain exerts a *cis* activity on the RdRP domain on CCHFV L.

In our hands, a reliable way to relieve the attenuated phenotype of the C40A L mutant was to overexpress conjugatable ISG15. Overexpression of ubiquitin, by contrast, had a negative influence, at least in human cells. As the protease-inactivated C40A OTU cannot cleave off these posttranslational modifications, it is conceivable that the binding of ISG15—conjugated to L or to another viral or cellular protein–is involved in activation of the L RdRP. For the wt L the subsequent cleavage of ISGylation may allow binding of ubiquitin as a counterbalancing negative regulator. Such a role of bound ubiquitin was proposed recently [31], and is confirmed by our observation that ubiquitin overexpression has a negative effect on L activity in human cells. These regulatory events may be indirectly connected to the IFN system via ISG15. Indeed, recombinant CCHFV with an L OTU unable to bind ubiquitin (Q16R) but able to bind ISG15 exhibited slightly increased viral RNA levels and IFN induction [30]. By contrast, a virus mutant that binds neither ubiquitin nor ISG15 (A129R) was reduced in RNA synthesis and did not induce IFN in most cell types [30]. Thus, ISG15 may stabilize a transcriptionally active conformation, or control the switch between genome replication and transcription. Alternatively, ISG15 binding may simply protect from a negative action by ubiquitin.

It will be challenging to disentangle the *cis*-activities of ubiquitin and ISG15 on RdRP regulation from the *trans*-activities on IFN induction as, at least in the case of live viruses, they influence each other. Using tc-VLP systems, inhibitors, IFN-deficient cells, and the full-length L protein, as we have done here may help to separate these different aspects of the viral OTU activity. OTU mutant viruses exhibit an increased induction of the immunostimulatory cytokine IFN [30]. Moreover, CCHFV tc-VLPs have shown promise as vaccine candidate [51], and C40A-mutation may increase their potency. Transient overexpression of ISG15 may become a tool to rescue lethal OTU mutant viruses and VLPs to use them as safe vaccine candidates.

## Supporting information

S1 Fig CCHFV L expression levels. Immunoblot analysis of lysates (left panels) and supernatants (right panels) from tc-VLP donor cells. Donor cells were transfected with the indicated plasmids, and the CCHFV N plasmid.
(TIF)

S2 Fig Absence of IFN induction by CCHFV tc-VLPs. Human A549 cells were infected with the IFN inducer virus RVFV-Ren (MOI 1; positive control) or the different tc-VLPs, and IFN induction was measured 24 h later by RT-qPCR as described in [43].
(TIF)

S3 Fig Unspecific reporter gene suppression by the overexpressed wt OTU domain. Cells were transfected with plasmids encoding components of the minireplicon systems of CCHFV (a) and RVFV (b) along with the FF-Luc control plasmid as described for Fig 4B or in [37], respectively.
(TIF)
