# Supplementary figures and images for: ISG15 overexpression compensates the defect of Crimean-Congo hemorrhagic fever virus polymerase bearing a protease-inactive ovarian tumor domain
Source: PLoS Negl Trop Dis. 2020 Sep 15;14(9):e0008610. doi: 10.1371/journal.pntd.0008610 (PMC7518590; doi:10.1371/journal.pntd.0008610)

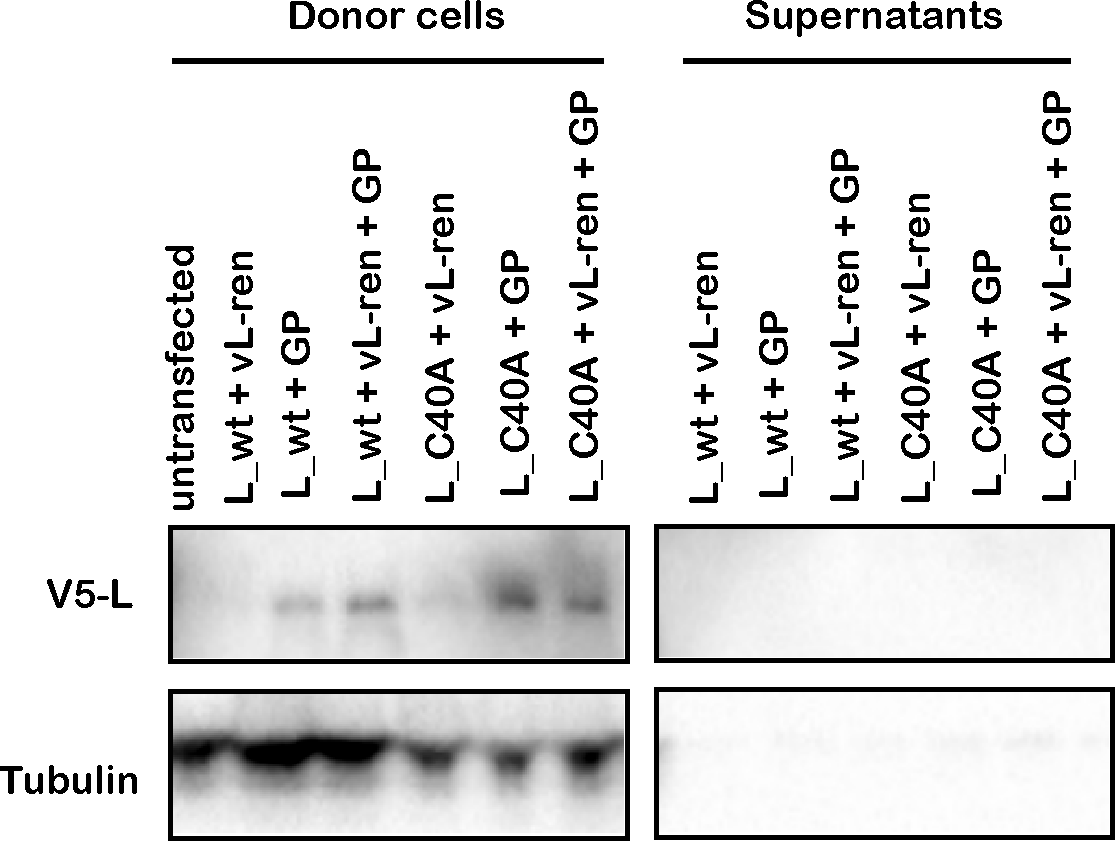

Supplement: S1 Fig — Immunoblot analysis of lysates (left panels) and supernatants (right panels) from tc-VLP donor cells. Donor cells were transfected with the indicated plasmids, and the CCHFV N plasmid. (TIF) [file pntd.0008610.s001.tif]

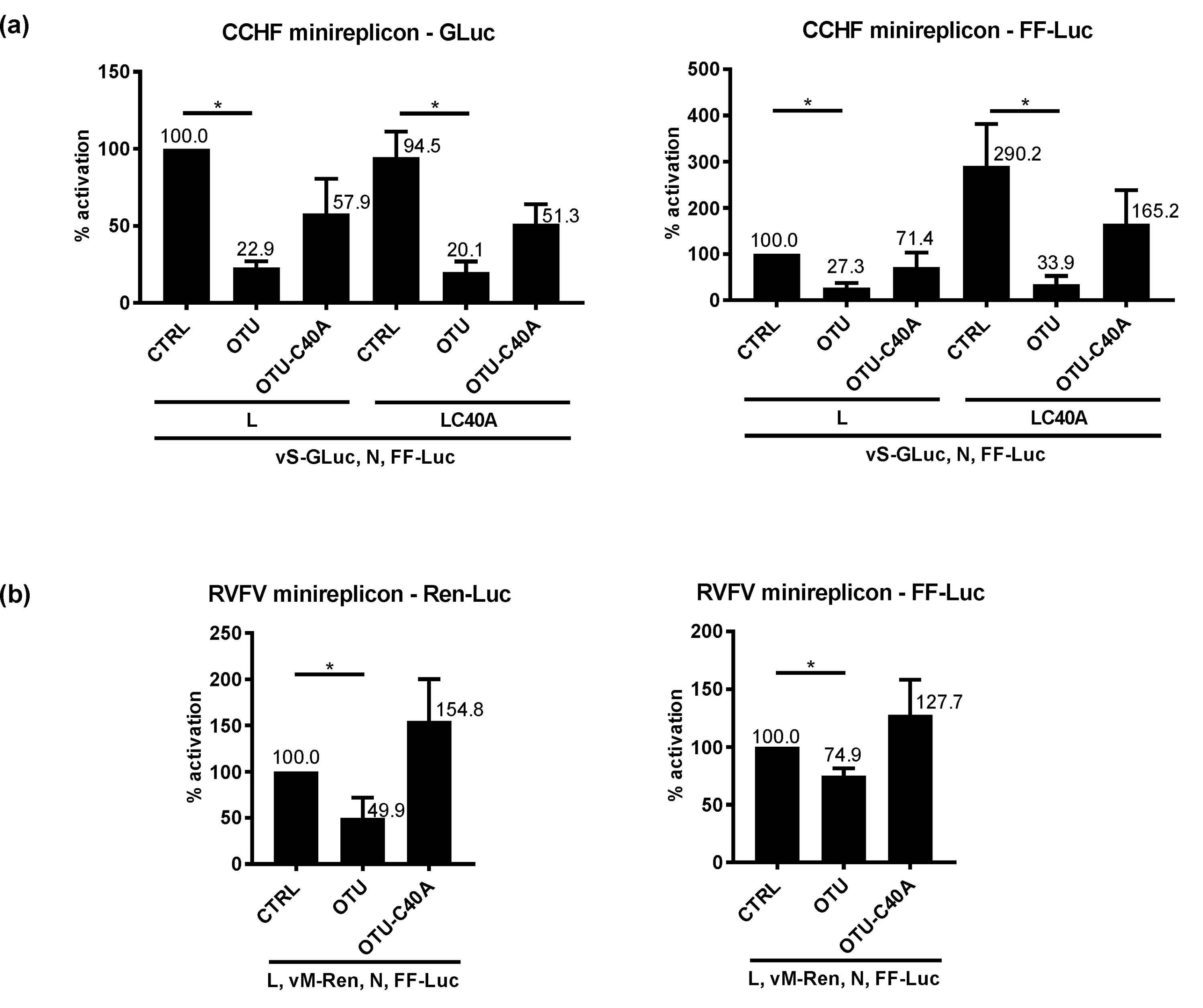

Supplement: S3 Fig — Cells were transfected with plasmids encoding components of the minireplicon systems of CCHFV (a) and RVFV (b) along with the FF-Luc control plasmid as described for Fig 4B or in [37], respectively. (TIF) [file pntd.0008610.s003.tif]
